# Open Fireplaces

**Published:** 1866-09

**Authors:** 


### OPEN FIREPLACES.

It is not possible to supply a pure warmth by any furnace ever invented, unless it simply heats water or air, out of which is given the caloric necessary to make a dwelling comfortable. But warming houses by steam, hot water, or hot air, costs, for an ordinary residence, about eight hundred dollars, which makes it impracticable—places this luxury wholly beyond four fifths of all the households in the land. That the heat which comes from any furnace through an ordinary register, although the coals are red-hot, is a sickening stench, can be demonstrated any moment in a winter's day; it is sending into a room an incessant stream of air, almost wholly divested of its oxygen, which is the element for which alone air is breathed at all; nor is this all—the oxygen has not only been abstracted, but sulphureted hydrogen and carboneted hydrogen, which are among the most noisome smells in nature—that of rotten eggs—replace the oxygen; and that such an atmosphere, steaming into our parlors, and dining-rooms, and chambers, can not be otherwise than most pernicious to health, only but an idiot can deny. Every year new patents are coming out, claiming to meet the failures of their predecessors, proving conclusively that all previous ones have been signal and lamentable failures.

It may be a more potent and convincing argument against the pestiferous effects of furnace heat, at least in the minds of some, that it ruins the furniture and the woodwork of all buildings into which it is introduced.

Open wood-fires, the most cheery and delightful of all modes of house-warming, are too expensive, and are exceedingly troublesome. The common open grates for coal are the next best, but they fail to give a comfortable heat in the coldest weather; they fail to keep the feet warm, which is the most important part of the body to be kept agreeably heated; and, in addition, the very instant the coal in the grate is touched, the whole room is filled with a fine dust, which settles on the paintings, the furniture, the carpets, and the very clothing in the drawers, making dingy the most polished surfaces, scratching the furniture and the gilding, and grinding out the carpets by the flinty dust.

But there is a method of warming houses, cheaper than grates and more efficient, giving almost none of their dust; incomparably less troublesome than wood-fires, while the heat is just as genial and quite as pure; the fire needs replenishing but once a day, never requires a poker, if properly attended to; gives very little dust, keeps the feet warm, and keeps before the eyes the cheery sight of a broad bed of burning, glowing coals. In short, it is a plan for warming houses, which has never, in all its points, been surpassed—has never been equaled. It is Dixon's low-down grate. It is believed that there is scarcely a single educated physician in Philadelphia, who owns the house he lives in, who is not supplied with one or more of these delightful luxuries. They cost from twenty-five dollars each and upward, and are placed in stead of an ordinary fireplace or grate in the course of a few hours.

Three fourths of the heat of a grate or fireplace goes up the chimney, and is wasted. Dixon's Philadelphia low-down grate, by a moderate extra expense, can be so arranged that all the ashes are conveyed into the cellar, and the otherwise wasted heat is saved to a considerable extent, and conveyed into the rooms above; not the heat of burning coals, but air is brought from out-doors, carried behind the chimney-back, heated without coming in contact with the coals, and is conveyed into the room above by an ordinary register, not in a sulphurous odor, but simply in the shape of pure air warmed, which is of inestimable value for sitting-rooms, chambers, and nurseries. We had one of these admirable contrivances put in our house in 1859, and every additional year only increases our appreciation of the luxury. This notice has been written without the knowledge of the manufacturer, and will surprise him as much as any one of our readers; but it would add so much to the health of families, both in town and country, whether they burn soft coal, anthracite, or common wood, for it is adapted to the consumption of any kind of solid fuel, that we feel constrained to bring it thus prominently forward, and the more fearlessly because we know whereof we affirm. To save us the expense, time, and trouble of answering letters of inquiry, our readers will please address T. W. Dixon, 1324 Chestnut street, Philadelphia, or his agents, Mead & Woodward, 37 Park Row, New-York City

